# Detection of SARS-CoV-2 and HHV-8 from a large pericardial effusion in an HIV-positive patient with COVID-19 and clinically diagnosed Kaposi sarcoma: a case report

**DOI:** 10.1186/s41182-022-00464-x

**Published:** 2022-09-24

**Authors:** Ryan R. Yanes, Greco Mark B. Malijan, Lyka Kymm Escora-Garcia, Stephanie Angel M. Ricafrente, Mary Jane Salazar, Shuichi Suzuki, Chris Smith, Koya Ariyoshi, Rontgene M. Solante, Edna M. Edrada, Kensuke Takahashi

**Affiliations:** 1 San Lazaro Hospital – Adult Infectious Diseases and Tropical Medicine Department, Manila, Philippines; 2 San Lazaro Hospital – Nagasaki University Collaborative Research Office, 3rd Floor Administration Building, San Lazaro Hospital, Quiricada Street, Sta. Cruz, 1003 Manila, Philippines; 3 Nagasaki University – School Tropical Medicine and Global Health, Nagasaki, Japan; 4 Department of Clinical Research – London School of Hygiene & Tropical Medicine, London, UK; 5 Institute of Tropical Medicine, Nagasaki University, Nagasaki, Japan; 6 Nagasaki University Hospital – Acute and Critical Care Unit, Nagasaki, Japan

**Keywords:** HIV, Kaposi sarcoma, HHV-8, COVID-19, SARS-CoV-2, Tuberculosis, Pericardial effusion, Co-infection, Opportunistic infections, Case report

## Abstract

**Background:**

Pericardial effusion is a late manifestation of HIV more commonly observed in individuals with depressed CD4 counts. Although *Mycobacterium tuberculosis* remains to be one of the most frequently identified pathogens in the pericardial fluid among people living with HIV, less commonly described etiologies include SARS-CoV-2 that causes coronavirus disease and human herpesvirus-8 which is associated with Kaposi sarcoma. Isolation of more than one pathogen in normally sterile sites remains challenging and rare. We report the first documentation of both SARS-CoV-2 and HHV-8 in the pericardial fluid.

**Case presentation:**

We present the case of a young man in his 20s with a recent history of clinically diagnosed pulmonary tuberculosis who was admitted for progressive dyspnea and cough. He had multiple violaceous cutaneous lesions on the face, neck, and trunk and diffused lymphadenopathies. He tested positive for SARS-CoV-2 on admission. The patient was clinically diagnosed with pneumonia, Kaposi sarcoma, and HIV/AIDS. Empiric broad spectrum antimicrobial regimen was subsequently initiated. HIV with low CD4 count was confirmed during hospitalization. Echocardiography revealed a large pericardial effusion, in impending cardiac tamponade. Frond-like fibrin strands, extending to the parietal pericardium, were also observed. Pericardiostomy yielded hemorrhagic, exudative effusion with lymphocytic predominance. SARS-CoV-2 and HHV-8 were detected in the pericardial fluid, and bacterial, fungal, and tuberculous studies were negative. The patient had clinical improvement after pericardial drainage. However, despite our best clinical care, he developed a nosocomial infection leading to clinical deterioration and death.

**Conclusion:**

Detection of SARS-CoV-2 and HHV-8 in the pericardial fluid is rare, and interpretation of their significance in clinical care is challenging. However, coronavirus disease and Kaposi sarcoma must be considered and adequately addressed in immunocompromised adults presenting with large pericardial effusion.

## Background

Pericardial effusion was a common manifestation among patients with HIV in the pre-antiretroviral therapy (ART) era [1]. In countries with widespread access to ART, the incidence of symptomatic pericardial disease has decreased dramatically [2]. However, it remains a significant problem in resource-limited settings and among patients with depressed CD4 and/or Acquired Immune Deficiency Syndrome (AIDS)-defining conditions.

Although *M. tuberculosis* is a highly prevalent cause of pericardial effusion in HIV and tuberculosis (TB)-endemic settings [3, 4], diverse etiologies have been reported including bacteria, fungi, and viruses [5–8]. HIV-associated lymphomas like Burkitt lymphoma and primary effusion lymphoma also result in pericardial effusion and tumors [9]. Majority of these malignancies are associated with Epstein–Barr virus (EBV) infection [10]. AIDS-related Kaposi sarcoma (KS), caused by Human Herpesvirus-8 (HHV-8), has been shown to involve the pericardium during autopsies. Recent reports suggest that SARS-CoV-2 also causes pericardial effusion presenting with myopericarditis, Takotsubo cardiomyopathy, and acute respiratory distress syndrome. However, COVID-19-associated pericardial effusion in an HIV-positive patient has not been reported to the best of our knowledge [11]. In this report, we present the first documentation of SARS-CoV-2 and HHV-8 detection in the pericardial fluid of a young HIV-positive patient.

## Case presentation

A male patient in his 20s was admitted to our hospital with a 2-week history of progressive dyspnea and cough. Five months prior to admission, he experienced non-productive cough accompanied by weight loss, night sweats, fever, and enlarged neck lymph nodes. He was clinically diagnosed with pulmonary TB. Intensive-phase therapy, comprising isoniazid–rifampicin–pyrazinamide–ethambutol combination, was initiated. He was adherent to treatment, and his symptoms improved after 2 months. Two-drug maintenance phase therapy was subsequently initiated. Two months prior to admission, the patient noticed multiple violaceous patches over the face, neck, and anterior chest. Eleven days prior to admission, he visited the outpatient department due to occurrence of cough, dyspnea, and fever. The patient was advised to undergo COVID-19 testing, but he refused due to fear of isolation. Worsening symptoms over the next week prompted emergency room (ER) visit. Further medical interview revealed history of unprotected sexual intercourse with multiple male partners beginning age 13. He was unvaccinated against COVID-19. The rest of the medical, social, and family history was unremarkable.

**Fig. 1:**
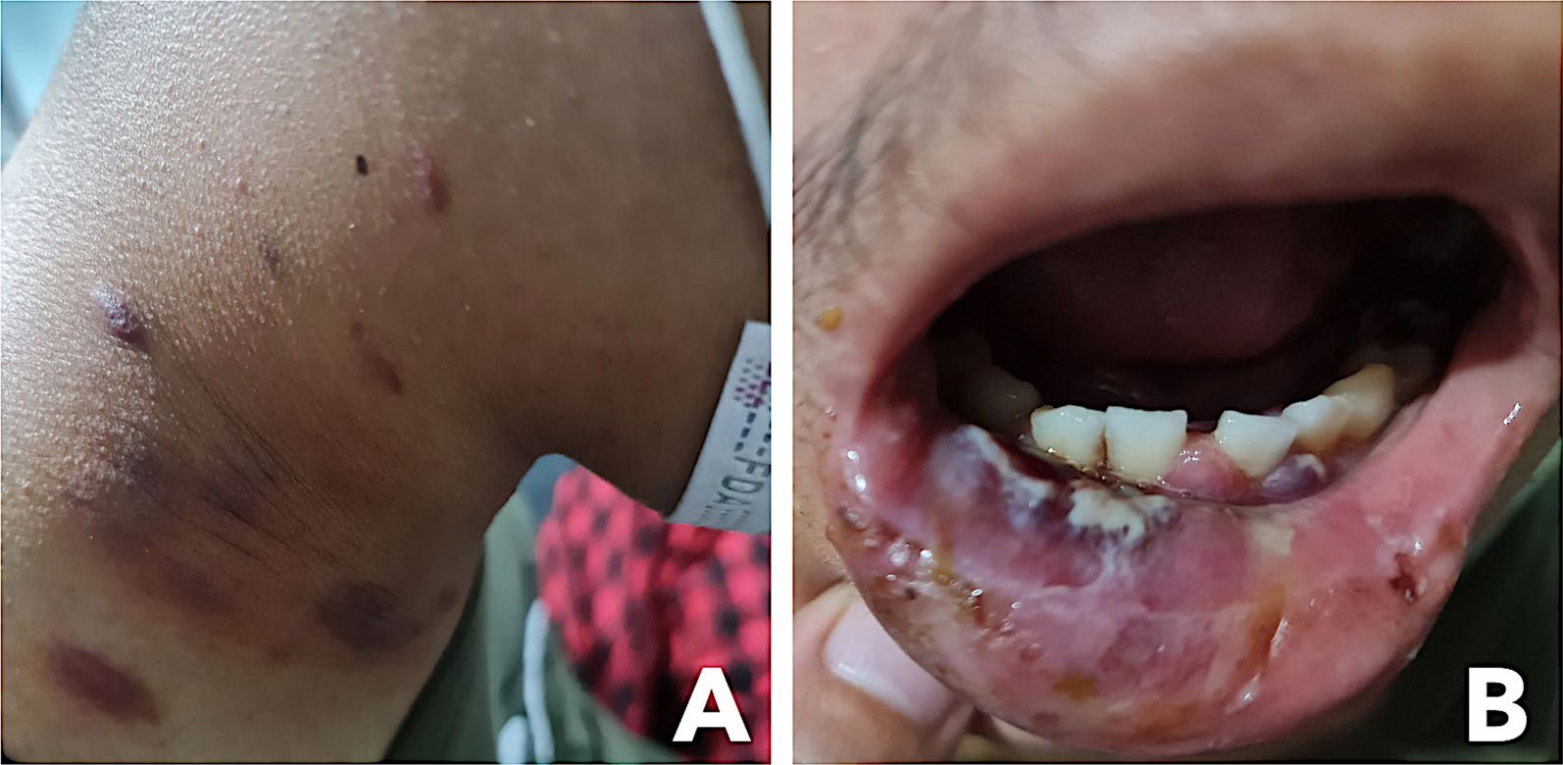
Multiple violaceous non-tender patches, plaques, and nodules on the neck (**A**) and purplish bruise-like mass and ulceration of the right lower lip (**B**)

At the ER, the patient was normotensive (110/70 mmHg), tachycardic (108 beats/min), tachypneic (28 cycles/minute), afebrile, and with oxygen saturation of 99% on room air. He was underweight, with a BMI of 18 kg/m^2^. Physical examination revealed well-defined, variably sized, violaceous, non-tender patches, plaques, and nodules on the face, neck, and trunk, measuring 3 cm at most. There was a well-defined, purplish mass with overlying ulceration on the right lower lip extending to the gingiva, with discrete purplish nodules on the lower alveolar ridge (Fig. 1). Coating patches of white slough were present on the mouth and oropharynx. Multiple matted, non-tender bilateral lymphadenopathies were palpable on the neck. There was no neck vein distention. Auscultation revealed crackles in the bilateral lung fields, adynamic precordium, and distinct heart sounds. Multiple non-tender inguinal lymphadenopathies with maximum size of 1 cm were palpable. The rest of the examination was within normal limits.

Due to the history of cough and dyspnea, the high-risk sexual practices, the presence of violaceous patches and nodules, the white slough in the oropharynx, and multiple lymphadenopathies, the primary impression was pneumonia in the immunocompromised host (COVID-19, *Pneumocystis jirovecii*, bacterial, tuberculosis), to consider HIV/AIDS with Kaposi sarcoma and candidiasis. The following medications were given empirically on admission: remdesivir, cotrimoxazole, ceftazidime, azithromycin, rifampicin, isoniazid, fluconazole, and prednisolone.

### Investigations and differential diagnoses

**Fig. 2:**
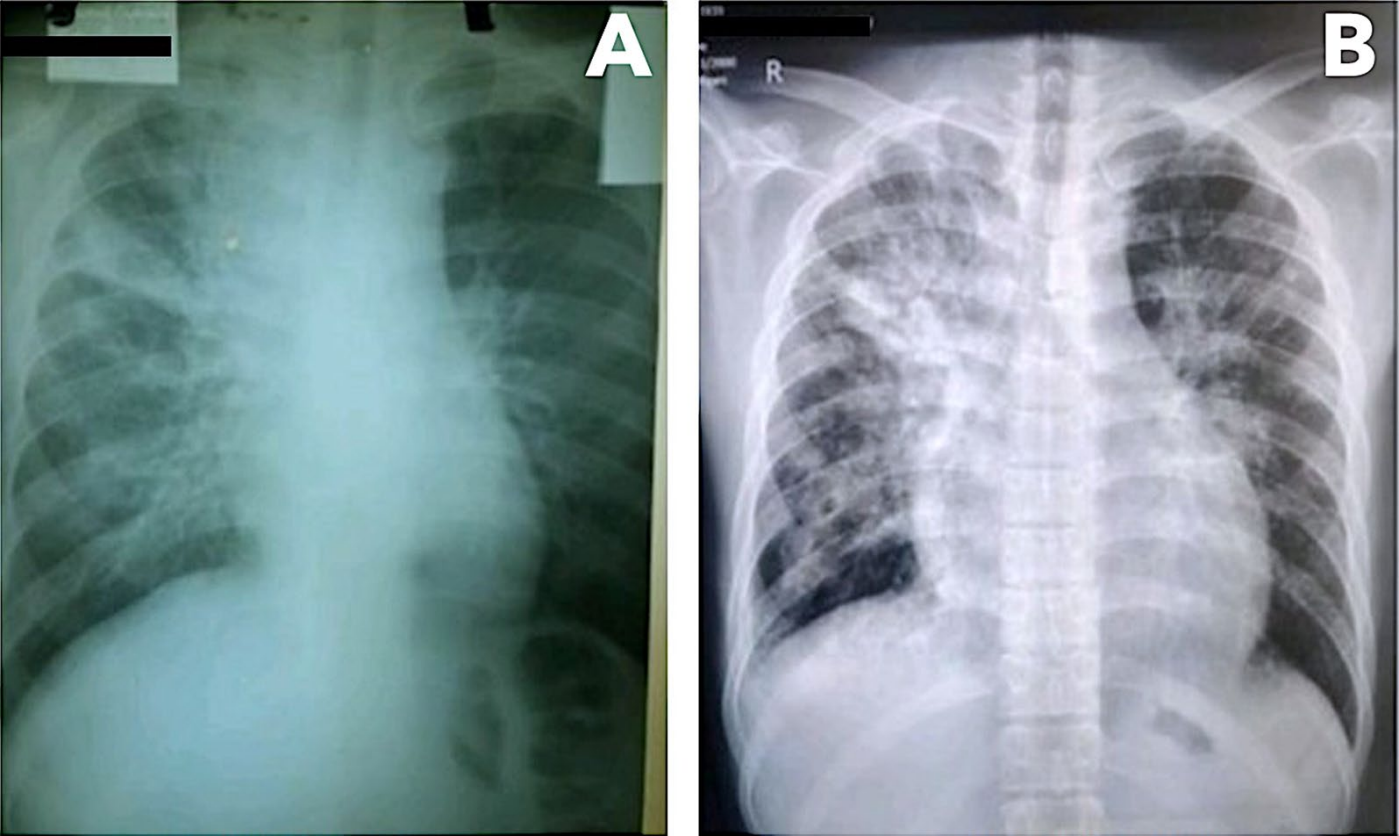
Chest radiographs taken 5 months prior to admission during initial diagnosis of tuberculosis (**A**) and taken on admission (**B**)

SARS-CoV-2 real-time reverse transcription polymerase chain reaction (RT-PCR) was positive on admission. The chest radiograph showed bilateral fibroreticular opacities, more pronounced on the right upper and middle lung fields. This finding was consistent with pulmonary TB (Fig. 2). Cardiac silhouette was visibly wider on admission radiograph compared to outpatient radiograph taken 5 months prior. Blood tests revealed slightly elevated C-reactive protein (18.71 µg/mL, normal value: < 10 µg/mL), normal procalcitonin (0.05 ng/mL, normal value: < 0.5 ng/mL), and normal lactate dehydrogenase (LDH, 391 U/L, normal range: 313–618 U/L). Kidney and liver function and cardiac troponins were within normal limits. Sputum acid-fast bacilli smear and GeneXpert® MTB/RIF assay were negative. Sputum bacterial culture later showed normal oral flora. Consequently, the empiric antibiotic therapy for bacterial pneumonia was discontinued.

HIV antibody/antigen test was positive, and confirmatory Western blot test was also positive. CD4 count was low (70 cells/µL), and HIV viral load was 784,000 copies/mL. Hepatitis B surface antigen was positive, and the remaining screening tests for sexually transmitted infections were negative.

Contrast chest and abdominal computed tomography (CT) scan revealed multiple nodules, air bronchograms, and surrounding ground-glass opacities in both lungs; prominent axillary lymph nodes; moderate pericardial effusion; and prominent mesenteric and peripancreatic lymph nodes, measuring 8 mm maximum.

**Fig. 3:**
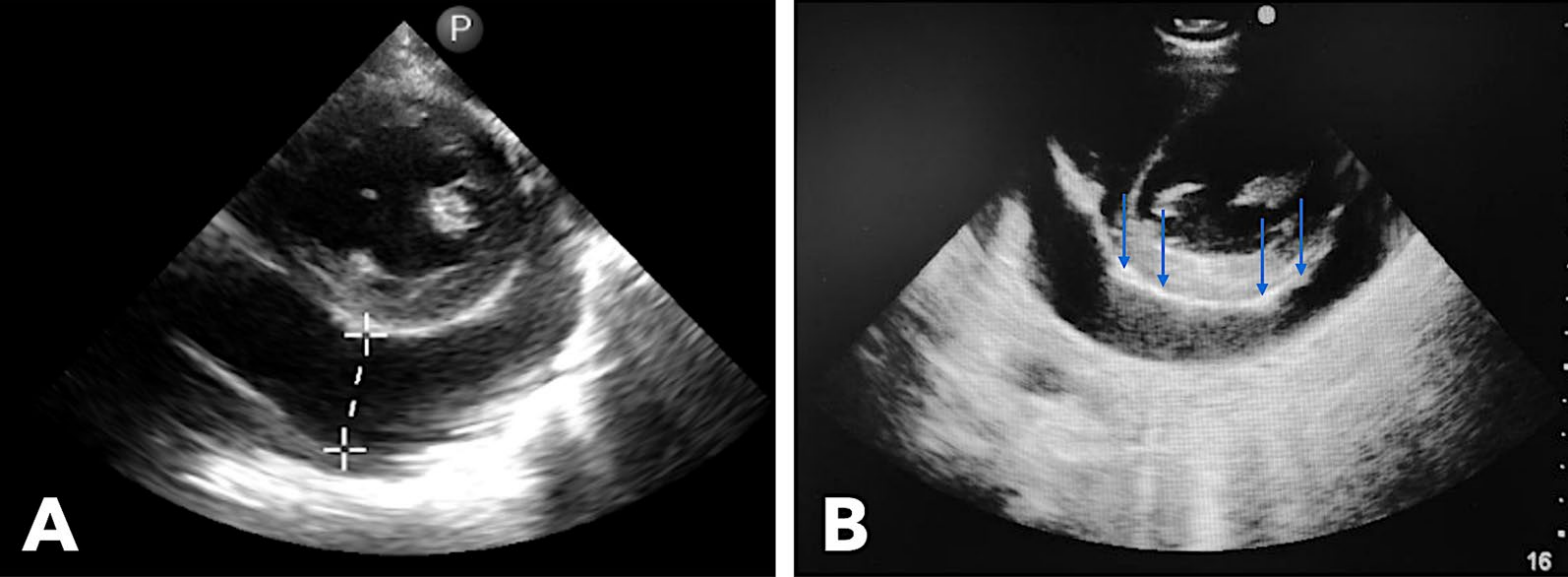
Echocardiography performed showing 3.1-cm echo-free space in the posterior pericardial space (**A**) and thickened pericardium with frond-like fibrinous projections (blue arrows) extending into the parietal pericardium (**B**)

Echocardiography showed large pericardial effusion, mild septal shift, and concentric left ventricular hypertrophy with preserved systolic function (Fig. 3). Frond-like fibrin strands were seen extending to the parietal pericardium.

Due to limited local available of more current anti-retroviral therapy regimen, lamivudine, tenofovir, and efavirenz single pill combination regimen was initiated on day 6. Moreover, dolutegravir-based regimen was not initiated due to potential enzyme-inducing effects of rifampicin, leading to reduced dolutegravir exposure [12]. In such cases, dosing may be increased, but single pill dolutegravir was not available locally.

Diagnostic and therapeutic pericardial drainage and tissue biopsy were contemplated, and transfer to a hospital with adequate facilities for post-cardiovascular surgical care was planned. However, logistic challenges associated with the patient’s COVID-19-positive status prevented the medical team from performing these interventions.

On days 9 to 18, the patient complained of worsening chest heaviness and dyspnea. There was no hypotension nor desaturation, and heart sounds were distinct on auscultation. Serial chest radiograph showed further increase in the size of the cardiac silhouette. He underwent emergency diagnostic and therapeutic pericardiostomy, and a drainage tube was placed, initially yielding 800 mL sanguineous, turbid fluid, which was sent for analysis.

From days 19 to 22, the patient was weaned off from oxygen support and had no episode of hemodynamic instability. Around 150–200 mL/day of serosanguinous pericardial fluid was drained. The effluent gradually decreased to < 10 mL/day.

Pericardial fluid analysis revealed hemorrhagic, exudative effusion with lymphocytic predominance (Table 1). Acid-fast bacilli were not isolated on Ziehl–Neelsen stain, and *M. tuberculosis* was not detected on GeneXpert® MTB/RIF assay. No fungal element was identified on the potassium hydroxide exam, and bacterial culture studies were negative. Cytology showed a chronic inflammatory pattern, and malignant cells were not identified. HHV-8 was detected on quantitative PCR (6,000 copies/mL). SARS-CoV-2 RdRP and E genes were detected on RT-PCR. Parallel nasopharyngeal/oropharyngeal (NP/OP) swab was also positive for SARS-CoV-2, albeit with lower cycle threshold (Ct) values. Multiplex PCR for common respiratory viruses including influenza A and B, adenovirus, bocavirus, parainfluenza, human metapneumovirus, respiratory syncytial virus, rhinovirus and seasonal coronavirus were all negative. Due to resource limitations, molecular tests for cardiotropic viruses, indirect TB detection methods, and tissue biopsy (pericardial, skin, and lymph node) were not performed.

**Table 1:** Pericardial fluid analysis

Assessment	Result
Color	Red
Clarity	Turbid
Red blood cell	Too numerous to count
White blood cell	182 cells/mm^3^
Neutrophils	12%
Lymphocytes	70%
Monocytes	16%
Acid-fast bacilli smear	Negative
Xpert MTB/Rif Assay	Not detected
Potassium hydroxide exam	Negative
Human Herpesvirus-8 qPCR	Detected (6 × 10^3^ copies/mL)
SARS-CoV-2 RT-PCR	Detected (RdRP gene *C*_*t*_ value—35.8, E gene *C*_*t*_ value—35.9)
Parallel SARS-CoV-2 RT-PCR for NP/OP swab	Detected (RdRP gene *C*_*t*_ value—18.6, E gene *C*_*t*_ value—33.7)
Conventional multiplex PCR for Influenza A, Influenza B, Parainfluenza Virus 1–4, Respiratory Syncytial Virus, Human Metapneumovirus, Rhinovirus, Human Coronavirus OC43, Human Coronavirus 229E	Not detected
Cytology	Chronic inflammatory pattern, no malignant cell identified
Bacterial culture studies	No growth

*C*_*t*_ value—cycle threshold value, *NP/OP* nasopharyngeal/oropharyngeal, *qPCR* quantitative polymerase chain reaction, *RT-PCR* real-time reverse transcriptase polymerase chain reaction

Based on the heavy TB burden in the Philippines [13] and the patient’s history of pulmonary TB, we highly considered tuberculous pericarditis in our case. Echocardiography showed typical, but not specific findings of frond-like fibrin projections from the visceral pericardium [3]. However, the patient’s adequate response to initial anti-TB treatment and the negative results of nucleic acid tests for *M. tuberculosis* on both sputum and pericardial fluid made tuberculous effusion less likely.

We could not completely rule out HIV-associated lymphomas due to lack of tissue biopsy. However, the absence of mediastinal tumors, hepatosplenomegaly, and other constitutional symptoms and the presence of normal LDH levels made lymphomas less likely.

Disseminated KS could present in any organ including the pericardium. Typical cutaneous lesions and lymphadenopathies and low CD4 count supported the diagnosis of KS. Although not diagnostically conclusive, the detection of HHV-8 suggested a precursor infection to the development of the disease.

We did not initially consider COVID-19 as the cause of the pericardial effusion due to the presence of other more likely etiologies. However, detection of SARS-CoV-2 in both the pericardial fluid and NP/OP swab made COVID-19-associated effusion probable.

### Outcome

**Fig. 4:**
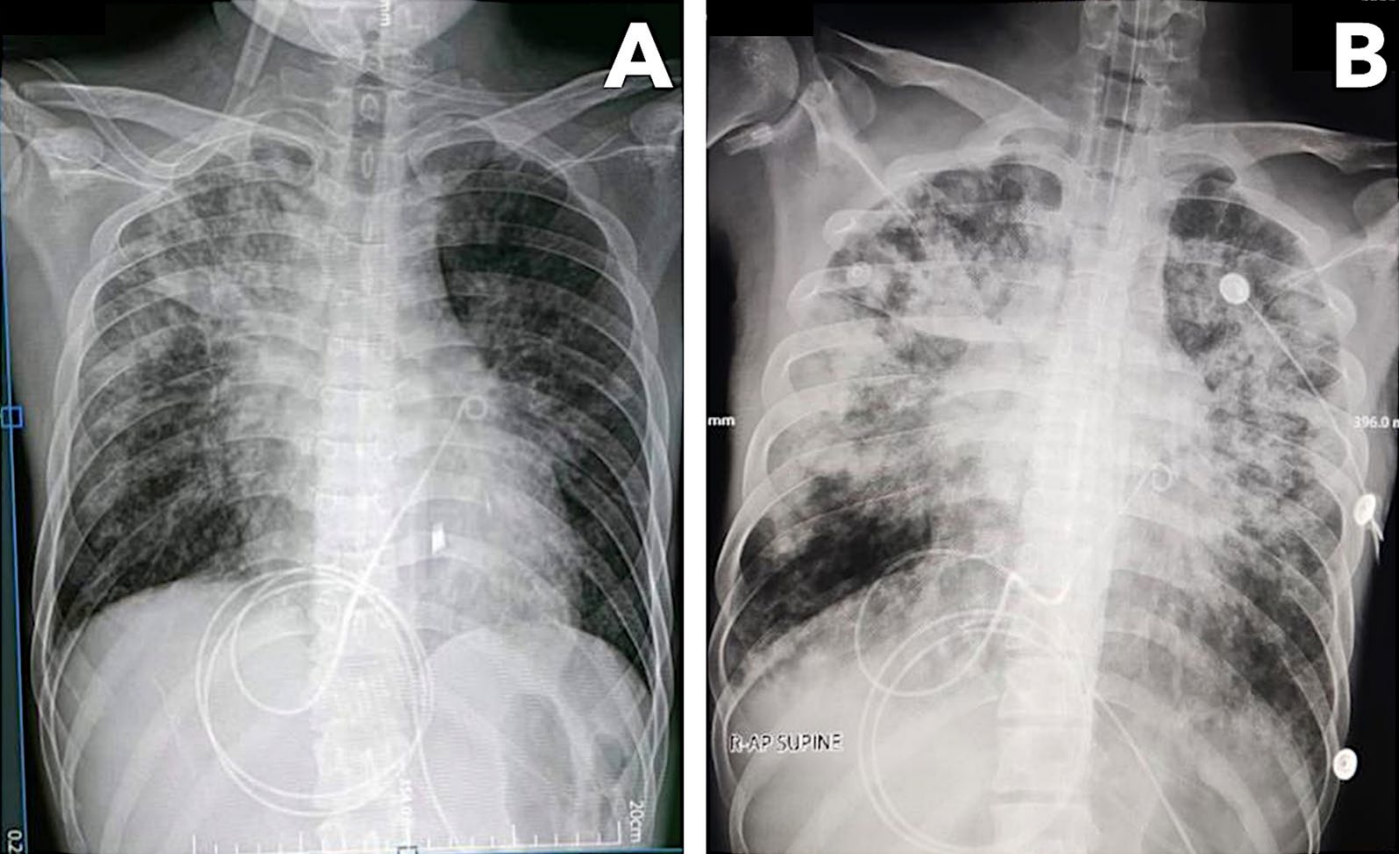
Chest radiographs taken after pericardiocentesis with visible reduction in the size of the cardiac silhouette (**A**) and taken 12 days after with progression of air bronchograms and fibroreticular infiltrates in both lung fields (**B**)

**Fig. 5:**
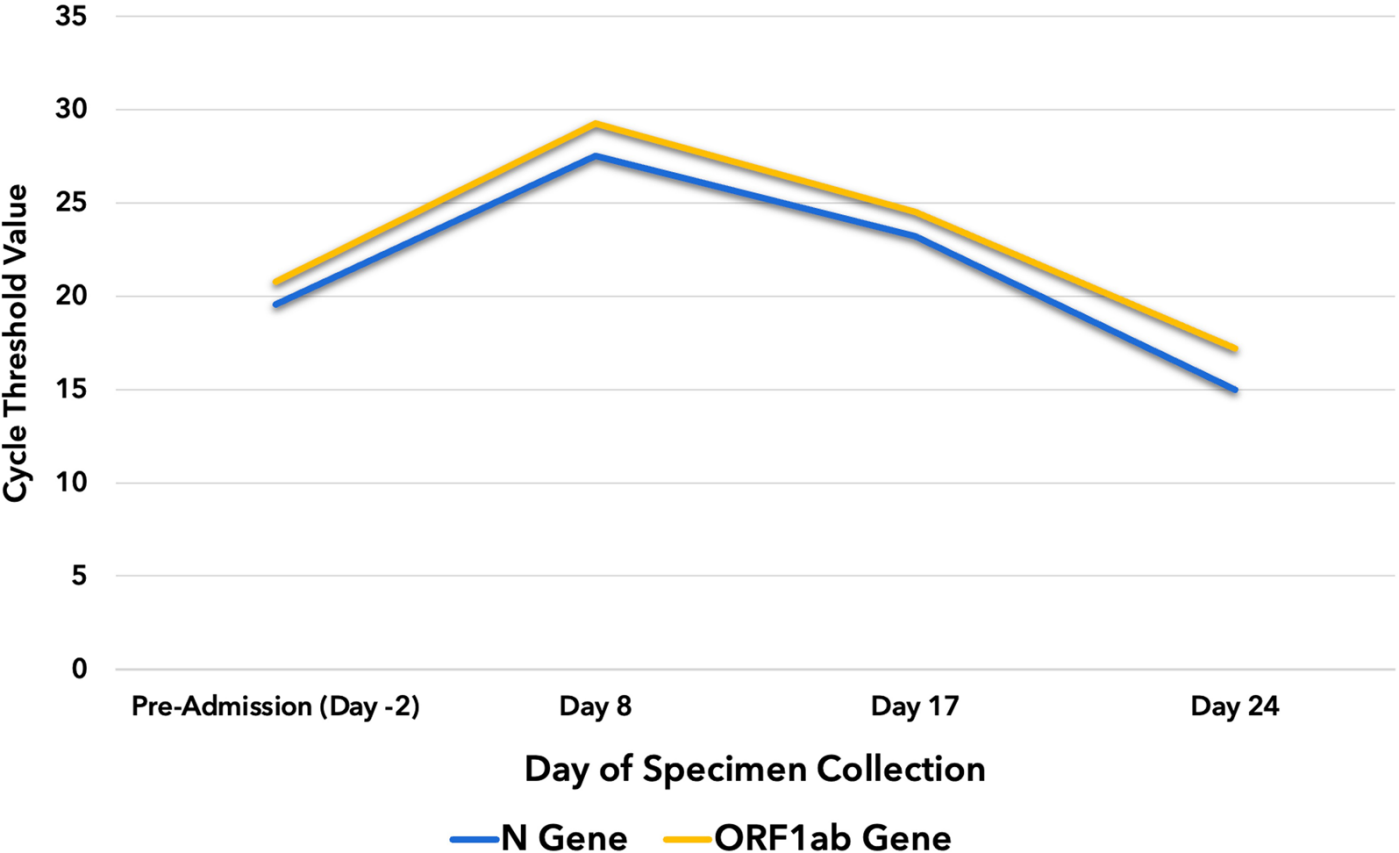
Changes in the cycle threshold values of SARS-CoV-2 genes detected during the patient’s hospitalization

Over the following week, the patient complained of gradually worsening dyspnea despite increasing oxygen support. He had coarse crackles on both lung fields and distinct heart sounds. Chest radiograph showed new bilateral reticular hazy opacities (Fig. 4). Chest CT scan revealed an interval progression of multifocal confluent and ground-glass opacities with air bronchograms in both lungs and minimal pericardial effusion. The patient was diagnosed with hospital acquired pneumonia, and empiric treatment with meropenem was added. On day 32, the patient developed acute respiratory failure and septic shock, prompting intubation, vasopressor initiation, and empiric vancomycin use. Despite maximal medical management, he died on day 35. The patient’s family did not consent for autopsy. Endotracheal aspirate culture showed extended spectrum beta-lactamase-producing *Enterobacter cloacae*. Throughout the hospitalization, the patient underwent SARS-CoV-2 tests every 7–10 days. N and ORF1ab genes were detected on four occasions (Fig. 5). Because of the declining Ct values coinciding with the patient’s deterioration, we explored the possibility of reinfection with a new variant. However, sequencing performed on the first and last specimens showed Delta variant (B1.617.2) congruently.

## Discussion and conclusions

Cardiac manifestations in SARS-CoV-2 infection are increasingly characterized [14, 15]. Detection of SARS-CoV-2 in the pericardial fluid has only been reported three times [16–18]. The reported cases involved immunocompetent elderly with prior history of cardiovascular diseases (i.e., two with non-ST segment elevation myocardial infarction and one with pulmonary embolism). However, our patient was an immunocompromised young adult without known cardiac comorbidity. The presence of large effusions with low estimated viral load was common among these four cases.

Current theories of pericardial injury in COVID-19 include direct invasion of cardiomyocytes and pericardium by SARS-CoV-2 and indirect injury through exaggerated inflammatory response [11]. Detection of SARS-CoV-2 in effusions support direct pericardial invasion, but the exact mechanism remains unclear. Furthermore, the limited number of publications reporting the detection of SARS-CoV-2 in pericardial fluid may be due to the small proportion of individuals who undergo pericardial drainage among COVID-19 patients with pericardial effusion [19].

Large pericardial effusions are considered to be a late sequela of COVID-19, presenting within 2–3 weeks of pulmonary symptoms and, in some instances, with undetectable virus on NP/OP samples [16, 20–23]. Various treatment modalities have been used, including pericardial drainage with or without colchicine [16–18, 21, 22] and uniportal video-assisted thoracoscopic surgery [24]. Similarly, our patient’s pericardial effusion was diagnosed after a 2-week history of new-onset cough and dyspnea. SARS-CoV-2 was detected in both NP/OP swab and pericardial fluid despite being sampled 5 weeks after initial symptom onset. Our findings support the evidence base for prolonged SARS-CoV-2 shedding among HIV-positive and other immunocompromised patients [25–27].

Detection of HHV-8 in the pericardial fluid is uncommon. The first reported case involved an HIV-seronegative patient with relapsing plasmacytic multicentric Castleman’s disease and Kaposi lesions on the toes [28]. Both pericardial and pleural fluid specimen were positive using single target PCR. HHV-8 is more commonly detected through immunohistochemistry staining in malignant cells including those isolated from pericardial fluid cytology [29]. Most cases are immunocompromised and/or HIV positive, like our patient [30].

Despite the negative sputum and pericardial fluid Xpert MTB/RIF assay and acid-fast bacilli smear, we could not completely rule out the occurrence of tuberculosis in our patient due to local disease epidemiology and clinical presentation. Nevertheless, detection of two or more pathogens in the pericardial fluid as in our case is extremely rare. Two reported cases involved *M. tuberculosis–Staphylococcus aureus* and *M. tuberculosis–Streptococcus pneumoniae* coinfections in HIV-positive patients who presented with acute purulent pericarditis and cardiac tamponade [31, 32].

Due to the significant number of likely etiologies for the patient’s clinical presentation, we attempted to be exhaustive in our management. Tissue biopsy was central in establishing the definitive diagnosis, but policies covering the COVID-19 status of patients, regardless of the duration since the first positive PCR test, severely prevented us from referring the patient to appropriate specialists. Because the patient was admitted in an infectious disease hospital, we deemed transfer to a more suitable facility for post-cardiovascular surgical care to be important. However, identified centers could not readily accommodate our referral due to lack of source isolation unit vacancy to address the patient’s multiple coinfections. Delayed diagnosis of HIV status and initiation of treatment likely contributed to the unfortunate outcome of the patient. The impact of social stigma associated with HIV in the Philippines remained apparent. Systemic delays and resource limitations likely contributed to the patient’s prolonged hospitalization and development of hospital acquired infection. Despite these challenges, we maximized the pericardial fluid analysis to guide our clinical management and to better characterize this rare case.

Detection of viruses like SARS-CoV-2 and HHV-8 in the pericardial fluid is rare, and interpretation of their significance in clinical care is challenging. Kaposi sarcoma should be considered in people living with HIV with characteristic cutaneous lesions, lymphadenopathies, and pericardial effusion. COVID-19 status-based hospital policies may prevent timely diagnostic and therapeutic interventions and disproportionately impact prolonged shedders including HIV patients and immunocompromised hosts. Finally, HIV status should be screened for young patients with TB in endemic countries.

## Data Availability

All data used in this article are available and may be requested from the corresponding author.

## Abbreviations

AIDS: Acquired immunodeficiency syndrome
ART: Antiretroviral therapy
COVID-19: Coronavirus disease-2019
Ct: Cycle threshold
CT: Computed tomography
EBV: Epstein–Barr virus
ER: Emergency room
HHV-8: Human herpesvirus-8
HIV: Human immunodeficiency virus
KS: Kaposi sarcoma
LDH: Lactate dehydrogenase
NP/OP: Nasopharyngeal/oropharyngeal
SARS-CoV-2: Severe acute respiratory syndrome coronavirus-2
TB: Tuberculosis
